# Cancer Survival in Adults in Spain: A Population-Based Study of the Spanish Network of Cancer Registries (REDECAN)

**DOI:** 10.3390/cancers14102441

**Published:** 2022-05-15

**Authors:** Marcela Guevara, Amaia Molinuevo, Diego Salmerón, Rafael Marcos-Gragera, Marià Carulla, María-Dolores Chirlaque, Marta Rodríguez Camblor, Araceli Alemán, Dolores Rojas, Ana Vizcaíno Batllés, Matilde Chico, Rosario Jiménez Chillarón, Arantza López de Munain, Visitación de Castro, Maria-José Sánchez, Enrique Ramalle-Gómara, Paula Franch, Jaume Galceran, Eva Ardanaz

**Affiliations:** 1 Navarra Public Health Institute, 31003 Pamplona, Spain; me.ardanaz.aicua@navarra.es; 2 Consortium for Biomedical Research in Epidemiology and Public Health (CIBERESP), 28029 Madrid, Spain; dsm@um.es (D.S.); rmarcos@iconcologia.net (R.M.-G.); mdolores.chirlaque@carm.es (M.-D.C.); mariajose.sanchez.easp@juntadeandalucia.es (M.-J.S.); 3 Navarra Institute for Health Research (IdiSNA), 31008 Pamplona, Spain; 4 Biodonostia Health Research Institute, 20014 San Sebastian, Spain; au-molinuevo@euskadi.eus; 5 Departamento de Ciencias Sociosanitarias, IMIB-Arrixaca, Universidad de Murcia, 30100 Murcia, Spain; 6 Epidemiology Unit and Girona Cancer Registry, Oncology Coordination Plan, Catalan Institute of Oncology, Department of Health, Government of Catalonia, 17007 Girona, Spain; 7 Descriptive Epidemiology, Genetics and Cancer Prevention Research Group, Girona Biomedical Research Institute (IdiBGi), 17190 Girona, Spain; 8 Faculty of Medicine, University of Girona, 17071 Girona, Spain; 9 Josep Carreras Leukemia Research Institute, 17003 Girona, Spain; 10 Tarragona Cancer Registry, Cancer Epidemiology and Prevention Service, Hospital Universitari Sant Joan de Reus, CatSalut, 43204 Reus, Spain; maria.carulla@salutsantjoan.cat (M.C.); jaume.galceran@salutsantjoan.cat (J.G.); 11 Pere Virgili Health Research Institute (IISPV), 43204 Reus, Spain; 12 Faculty of Medicine and Health Sciences, Rovira i Virgili University, 43204 Reus, Spain; 13 Department of Epidemiology, Murcia Regional Health Council, IMIB-Arrixaca, 30008 Murcia, Spain; 14 Public Health Directorate of Asturias, 33006 Oviedo, Spain; marta.rodriguezcamblor@asturias.org; 15 Canary Islands Cancer Registry, Public Health Directorate, Canary Health Service, 35003 Las Palmas de Gran Canaria, Spain; maleher@gobiernodecanarias.org (A.A.); mrojmar@gobiernodecanarias.org (D.R.); 16 Castellón Cancer Registry, Public Health Directorate, General Health Department, Generalitat Valenciana, 46020 Valencia, Spain; vizcaino_ana@gva.es; 17 Ciudad Real Cancer Registry, Health and Social Welfare Authority, Castile-La Mancha, 13071 Ciudad Real, Spain; matildec@jccm.es; 18 Cuenca Cancer Registry, Health and Social Welfare Authority, Castile-La Mancha, 16071 Cuenca, Spain; rjimenez@jccm.es; 19 Basque Country Cancer Registry, Health Department, 01010 Vitoria, Spain; arantza-lopez@euskadi.eus (A.L.d.M.); v-castro@euskadi.eus (V.d.C.); 20 Escuela Andaluza de Salud Pública (EASP), 18011 Granada, Spain; 21 Instituto de Investigación Biosanitaria ibs.GRANADA, 18012 Granada, Spain; 22 Department of Preventive Medicine and Public Health, University of Granada, 18071 Granada, Spain; 23 Department of Epidemiology and Prevention, La Rioja Regional Health Authority, 26071 Logroño, Spain; eramalle@larioja.org; 24 Balearic Islands Health Research Institute (IdISBa), Illes Balears, 07120 Palma, Spain; pfranch@dgsanita.caib.es; 25 Mallorca Cancer Registry, Balearic Islands Public Health Department, 07010 Palma, Spain

**Keywords:** cancer, survival, prognosis, epidemiology, population-based study, cancer registries

## Abstract

**Simple Summary:**

We studied cancer survival and its trends in adult patients in Spain. We included more than 600,000 patients with primary cancer diagnosed during 2002–2013 and followed them up to 2015. The study provides cancer survival estimates up to five years after diagnosis by sex and age for 29 cancer groups. We found survival improvements for most cancer groups from 2002–2007 to 2008–2013, although with differences by age, being greater for patients younger than 75 years than for older patients. The persistent poor prognosis for some cancers emphasizes the need to reinforce actions along the cancer continuum, from primary prevention to early diagnosis, optimal treatment, and supportive care. Further examination of possible sociodemographic inequalities is warranted.

**Abstract:**

The assessment of cancer survival at the population level is essential for monitoring progress in cancer control. We aimed to assess cancer survival and its trends in adults in Spain. Individual records of 601,250 adults with primary cancer diagnosed during 2002–2013 and followed up to 2015 were included from 13 population-based cancer registries. We estimated net survival up to five years after diagnosis and analyzed absolute changes between 2002–2007 and 2008–2013. Estimates were age-standardized. Analyses were performed for 29 cancer groups, by age and sex. Overall, age-standardized five-year net survival was higher in women (61.7%, 95% CI 61.4–62.1%) than in men (55.3%, 95% CI 55.0–55.6%), and ranged by cancer from 7.2% (pancreas) to 89.6% (prostate) in men, and from 10.0% (pancreas) to 93.1% (thyroid) in women in the last period. Survival declined with age, showing different patterns by cancer. Between both periods, age-standardized five-year net survival increased overall by 3.3% (95% CI 3.0–3.7%) in men and 2.5% (95% CI 2.0–3.0%) in women, and for most cancer groups. Improvements were greater in patients younger than 75 years than in older patients. Chronic myeloid leukemia and myeloma showed the largest increases. Among the most common malignancies, the greatest absolute increases in survival were observed for colon (5.0%, 95% CI 4.0–6.0%) and rectal cancers (4.5%, 95% CI 3.2–5.9%). Survival improved even for some cancers with poor prognosis (pancreas, esophagus, lung, liver, and brain cancer). Further investigation of possible sociodemographic inequalities is warranted. This study contributes to the evaluation of cancer control and health services’ effectiveness.

## 1. Introduction

Cancer is a major cause of morbidity and mortality globally. Recent World Health Organization (WHO) estimates showed that cancer is the first leading cause of premature death (before 70 years of age) in 57 of 183 countries, including Spain, and ranks second in a further 55 countries [1]. In 2020 in Spain, with projected 280,000 new cancer cases [2], and 113,000 cancer deaths, the disease remains the first cause of death in men and the second in women, accounting for 27% and 19% of all deaths, respectively [3].

Population-based cancer survival, in addition to being a fundamental measure to describe the prognosis of cancer patients, is an indicator of the overall performance of health services across the patient pathway, from early detection and diagnosis to treatment and follow-up. Moreover, wide regional and international differences have been revealed, largely reflecting socioeconomic inequalities and differences in the effectiveness of health systems [4,5,6]. Hence, cancer survival estimates are of great importance to patients, clinicians, public health professionals, and policymakers.

The CONCORD-3 Programme updated the worldwide 5-year survival estimates to 2014 (2013 for Spanish adults) for 18 groups of cancers, however it did not provide data by sex nor by age group at the country level [4]. The latest specific study on survival of cancer patients in Spain, performed by the Spanish Network of Cancer Registries (REDECAN), provided results for patients diagnosed up to 2007 [7], therefore, detailed and more up-to-date estimates are needed. 

The aims of this study were (1) to provide cancer survival estimates for adult patients, diagnosed during 2008–2013 in Spain, overall and for 29 cancer groups by sex and age group, and (2) to assess survival trends between 2002–2007 and 2008–2013.

## 2. Materials and Methods

### 2.1. Study Design and Population

We conducted an observational study using data from 13 population-based cancer registries (Asturias, Canary Islands, Castellón, Ciudad Real, Cuenca, Basque Country, Girona, Granada, La Rioja, Mallorca, Murcia, Navarra and Tarragona), covering more than 12 million inhabitants that represent ~26% of the Spanish population of 2013. All the participating cancer registries follow standard registration procedures and have met the high standards of comparability, completeness and validity for inclusion in the series Cancer Incidence in Five Continents, published quinquennially by the International Agency for Research on Cancer (IARC) [8]. Most registries provided data for the entire study period and all included at least three years of each comparison period (Supplementary Table S1). 

We selected the cancer registration records for adults (aged 15–99 years) diagnosed between 2002 and 2013 with a primary malignant neoplasm, except non-melanoma skin cancer. Tumor anatomical site (topography) and morphology were coded according to the International Classification of Disease for Oncology, 3rd edition (ICD-O-3) [9]. The international rules for registering multiple primary neoplasms were applied [10], to enable comparison of the results with other studies. Patients who had more than one primary cancer were included in the analyses for each cancer. Only invasive malignancies (ICD-O-3 behaviour code 3) were included, except for the bladder, for which tumors with uncertain or borderline malignancy and in situ (behavior codes 1 and 2) were also included, to ensure comparability. Cases known to the registry only through the death certificate (DCO) and cases diagnosed at autopsy were excluded from the analyses, since their survival time was unknown. 

Analyses were performed for all cancers combined and separately for 29 cancer groups that jointly represent more than 90% of all cancer cases. Cancer groups were defined by topography and morphology. The WHO classification and the HAEMACARE guidelines were followed for grouping the hematological malignancies [11,12]. For presentation, the ICD-O-3 codes were converted to the International Classification of Diseases, Tenth Revision (ICD-10) codes by using the software IARCcrg Tools v2.13 (IARC, Lyon, France). Table 1 shows the cancer groups analyzed and the respective ICD-10 codes.

### 2.2. Follow-Up for Vital Status

Vital status follow-up until the end of 2015 was carried out using multiple sources of information in all cancer registries, such as the regional mortality registries, National Death Index, social security database, hospital and primary care records and population censuses, as needed and available in each registry. 

### 2.3. Quality Control

In addition to the quality controls performed in each cancer registry, extensive data quality checks were undertaken on the joint database, including those proposed by EUROCARE-5 [13], using IARCcrg Tools v2.13 (IARC, Lyon, France), JRC-ENCR Quality Check Software v1.8.1 (ENCR, Ispra, Italy), and automated ad-hoc checks. The records with definite or possible errors were returned to the registries for verification, correction and resubmission.

We assessed the proportions of cases microscopically verified and cases with non-specific morphology. We also evaluated the percentage of patients lost to follow-up, defined as cases censored alive before five years from diagnosis unless they were censored due to the end of follow-up (31 December 2015). 

### 2.4. Statistical Analyses

We estimated observed and net survival up to five years after diagnosis, with 95% confidence intervals (CIs), for adults diagnosed during 2002 through 2007 and 2008 through 2013, by sex and age group for each cancer group. The Kaplan-Meier and Pohar-Perme methods were used to obtain observed and net survival estimates, respectively [14]. Net survival is the probability of being alive at a certain time following diagnosis after controlling for other causes of death (background mortality). To control for background mortality, we built life tables of all-cause death rates in the general population for each province, by single year of age, sex, and calendar year of death. The source of population and death data was the Spanish National Institute of Statistics. Life tables were interpolated and smoothed by the Elandt-Johnson method [15]. The cohort approach was adopted for patients diagnosed in 2002–2007 and the period approach for those diagnosed in 2008–2013 [16], given that follow-up was carried out up to 2015, thus a complete 5-year follow-up was not available for all the cases incident during the latter period. 

To allow comparisons over time and with other studies, net survival estimates were age-standardized using the cancer-specific weights from the International Cancer Survival Standards (ICSS) [17]. The ICSS age groups are defined as 15–44, 45–54, 55–64, 65–74 and ≥75 years, with a variation for prostate cancer patients (15–54, 55–64, 65–74, 75–84 and ≥85). We present age-standardized net survival (ASNS) at 1, 3 and 5 years post-diagnosis and at 5 years post-diagnosis, conditional on having survived 1 year. The latter was obtained by restricting the analysis to patients who survived at least 1 year. The 95% CIs were calculated from the standard errors using the Greenwood formula [18].

Absolute changes in survival between both periods were measured as the arithmetic difference between ASNS estimates (ASNS *_2008–2013_—*ASNS *_2002–2007_*), for instance, a change in survival from 10% to 15% is reported as an increase of 5% (not 50%). A change was considered statistically significant if the 95% CI did not include zero. Trends were analyzed for all ages by sex and for two broad age groups (<75 and ≥75 years at diagnosis).

The analyses were performed using STATA 15.1 (Stata Corporation, College Station, TX, USA), and specifically the *stns* command to estimate net survival [19].

## 3. Results

### 3.1. Study Population and Quality Indicators

A total of 601,250 adult patients diagnosed with cancer (except non-melanoma skin cancer) over the 12-year period were included in the study, representing 98.1% of the eligible cases. The remaining 1.9% (11,723 cases) were excluded because they were DCO or diagnosed at autopsy. Men accounted for 60.4% (363,144 cases) of the study population and the mean age ±SD was 68 ±13 and 65 ±16 years in men and women, respectively. The number of cases and data quality indicators by cancer group are shown in Table 1. A high proportion of diagnoses were microscopically verified: 91% overall and ≥80% in all cancer groups with the exception of liver, pancreas, gallbladder and brain cancers. About 9% of tumors were recorded with a non-specific morphology, and only 0.23% of patients were lost to follow-up. 

### 3.2. Cancer Survival in Patients Diagnosed in 2008–2013

The 5-year observed survival (5y-OS) and net survival (5y-NS) estimates in patients diagnosed during the last six-year period (2008–2013) are shown in Table 2. For all cancers combined, 5y-OS was 47.4% and 57.4% in men and women, respectively; and 5y-NS, i.e., survival after controlling for other causes of death, was 54.3% and 62.0% in men and women, respectively. By cancer group, the lowest 5y-NS (<10%) was found for pancreatic cancer in men and women while the highest (≥95%) was observed for testicular cancer in men and thyroid cancer in women. The largest differences between 5y-OS and 5y-NS were found for prostate cancer (78% vs. 90%) and chronic lymphoid leukemia (62.5% vs. 73.6% in both sexes), while the smallest differences were seen for brain cancer (13.7% vs. 14.1% in both sexes).

**Figure 1:**
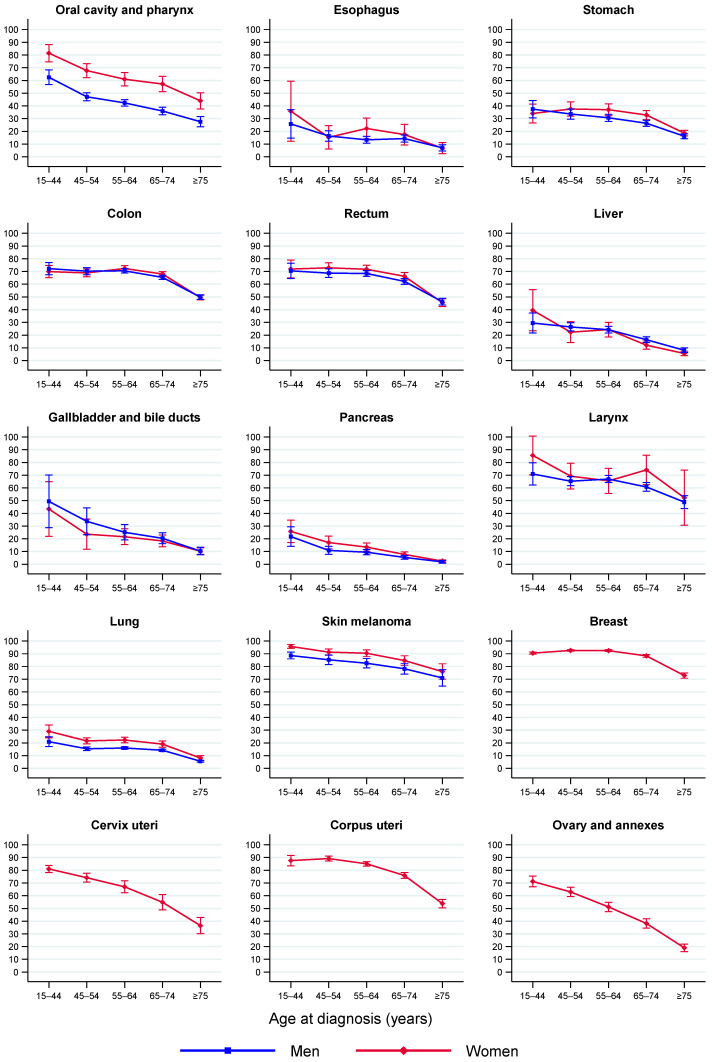

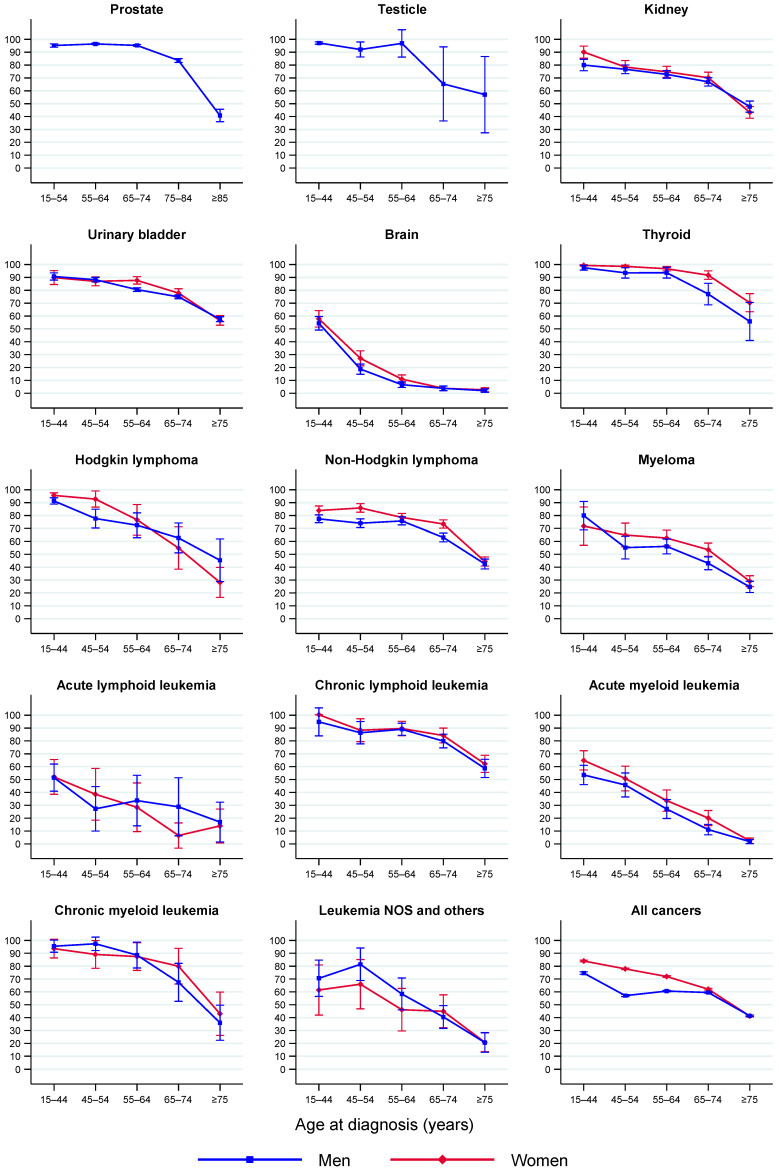
Five-year net survival by age group and sex in adult patients diagnosed with cancer in Spain in 2008–2013. Error bars are 95% CIs. The “all cancers” category excludes non-melanoma skin cancer. Abbreviations: NOS, not otherwise specified.

Age-specific 5y-NS estimates by sex for each cancer group in 2008–2013 are shown in Figure 1 and are detailed for men, women and both sexes in Supplementary Table S2. Survival declined with age at diagnosis, although for some cancers it occurred only after a certain age, for instance, for colon, rectal, breast and testicular cancers after the age of 65 years and for prostate cancer after 75 years. The downward trend over age was steeper for chronic myeloid leukemia, myeloma, prostate and brain cancer in men and for Hodgkin lymphoma, acute myeloid leukemia, brain and ovarian cancer in women. 

Table 3 shows the ASNS at one, three, and five years following diagnosis, and at five years following diagnosis conditional on having survived 1 year (5|1 years). The ASNS at the first year was 74.7% (95% CI 74.6–74.9%) overall and varied by cancer group from <40% for pancreas, esophagus and lung cancers to ≥95% for skin melanoma, female breast and prostate cancers. We observed a 5y-ASNS of 58.2% (95% CI 58.0–58.4%) overall, ranging from <20% for the pancreas, esophagus, lung, and liver cancer to ≥80% for cancers of the thyroid, prostate, testicle, skin melanoma, female breast, and Hodgkin lymphoma. The 5|1y-ASNS was 77.1% (95% CI 76.8–77.3%) for all cancers combined, and it was more than 20 percentage points higher than the 5y-ASNS for cancer of the esophagus, stomach, gallbladder and acute lymphoid leukemia.

The 5y-ASNS in 2008–2013 was higher in women than in men overall and for 12 cancer groups: oral cavity and pharynx, stomach, rectum, pancreas, lung, skin melanoma, urinary bladder, brain, thyroid, non-Hodgkin lymphoma, myeloma and acute myeloid leukemia (Table 4). For the remaining cancer groups, the 5y-ASNS was similar in both sexes. The most pronounced female survival advantage was found for cancer of the oral cavity and pharynx, with 5y-ASNS of 38.2% (95% CI 36.6–39.9%) in men vs. 57.2% (95% CI 54.4–60.2%) in women.

### 3.3. Survival Trends between 2002–2007 and 2008–2013

**Figure 2:**
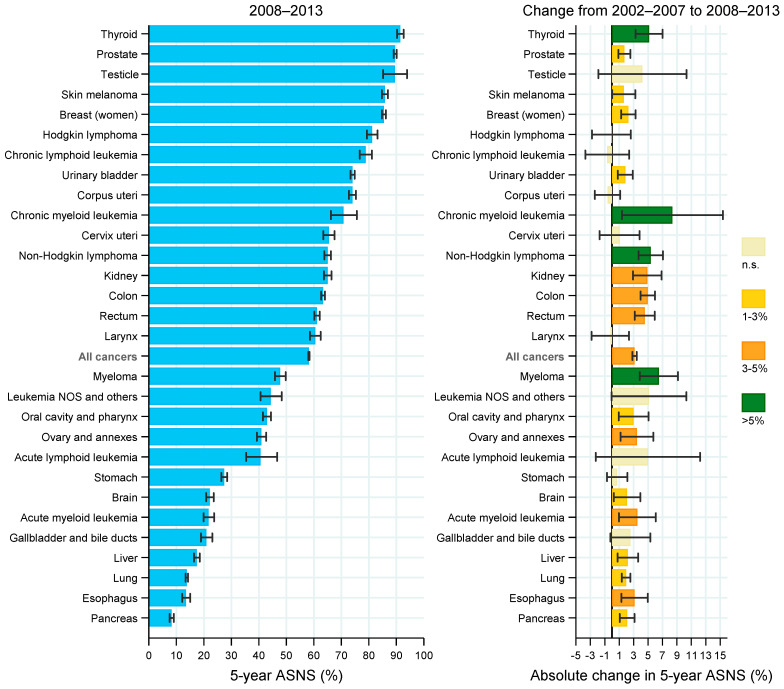
Five-year age-standardized net survival by cancer group in patients diagnosed with cancer in Spain in 2008–2013 (**left panel**) and absolute change from 2002–2007 to 2008–2013 (**right panel**). Error bars are 95% CIs. Abbreviations: ASNS, age-standardized net survival; n.s., non-statistically significant change (i.e., 95% CI includes zero).

In both sexes together, there were significant increases in survival overall and for most of the cancer groups between 2002–2007 and 2008–2013, as shown in Figure 2. The 5y-ASNS increased by more than 5% in patients with thyroid cancer, chronic myeloid leukemia, non-Hodgkin lymphoma and myeloma. Absolute increases of 3–5% were observed for kidney, colon, rectum, ovary and annexes, acute myeloid leukemia and esophageal cancer. In addition, increases of 1–3% in the 5y-ASNS were found in patients with cancer of the prostate, skin melanoma, female breast, urinary bladder, oral cavity and pharynx, brain, liver, lung, and pancreas. The trend was stable for the remaining cancer groups.

The trend analysis by sex (Table 4) showed that the 5y-ASNS for all cancers combined increased between the two periods from 52.0% (95% CI 51.7–52.2%) to 55.3% (95% CI 55.0–55.6%) in men, and from 59.2% (95% CI 58.9–59.6%) to 61.7% (95% CI 61.4–62.1%) in women. The increases in 5y-ASNS for cancer of the colon, rectum, pancreas, kidney, urinary bladder, thyroid, non-Hodgkin lymphoma and myeloma were observed in both men and women. An upward trend in 5y-ASNS for cancer of the esophagus, liver, lung, skin melanoma, and chronic myeloid leukemia was found only in men, while it remained stable in women. Increased 5y-ASNS for cancer of the oral cavity and pharynx, brain, acute myeloid leukemia, and the group of leukemia not otherwise specified (NOS) and others was observed only in women, whereas it did not change in men. There were no significant decreases in survival in men or women for any cancer group.

Among the most common cancers, the largest increases in the 5y-ASNS were found for colon cancer, from 57.5% to 63.1% in men and from 59.8% to 63.9% in women, and for rectal cancer, from 55.8% to 60.4% in men and from 58.1% to 62.7% in women. In addition, the 5y-ASNS for prostate cancer increased from 87.8% to 89.6%, and for female breast cancer from 83.2% to 85.5%. For patients with lung cancer the figures moved from 11.2% to 12.7% in men and from 16.2% to 17.6% in women, although the change in women was not statistically significant. Increases in the 5y-ASNS were also seen for urinary bladder tumors in men (by 1.6%) and women (by 3.0%). However, for corpus uteri cancer the 5y-ASNS remained unchanged between the first and second periods, 74.6% vs. 74.0%, respectively (Table 4).

The results of the trend analysis according to age group at diagnosis are shown in Table 5. In patients younger than 75 years, the 5y-ASNS increased for all cancers combined (by 4.5%, 95% CI 4.1 to 4.8%) and for 20 cancer groups. By contrast, in patients aged 75 years and older, the 5y-ASNS was stable for all cancers combined (absolute change of −0.1%, 95% CI −0.8 to 0.5%) and increased only for five cancer groups (colon, thyroid, non-Hodgkin lymphoma, myeloma, and acute lymphoid leukemia). Although an upward trend was observed mainly in patients younger than 75 years, in the older age group there were larger increases for thyroid cancer and acute lymphoid leukemia.

## 4. Discussion

The present study provides a comprehensive overview of cancer survival in Spain using the most up-to-date data from population-based cancer registries. More than 600,000 adult patients diagnosed until 2013 and followed up to 2015 were included. This study updates a previous publication incorporating six additional years of incidence [7], and also expands coverage as more registries were able to contribute data, reaching more than a quarter of the Spanish population. 

Survival estimates vary widely by cancer group [4,5,7]. The range of variation we found is broadly consistent with that reported by other population-based studies [4,20,21,22]. Very good prognosis (5y-ASNS ≥ 80%) was observed for cancers of the thyroid, prostate, testicle, female breast, skin melanoma and Hodgkin lymphoma. According to incidence estimates [23], these cancers represent 29% of all cancer cases in Spain, and account for a greater proportion in women than in men, 34% vs. 26%, respectively. Other frequent cancers, including those of the colon, rectum, urinary bladder and corpus uteri, had good prognosis (5y-ASNS 60–79%). However, cancers of the pancreas, esophagus, lung and liver showed 5y-ASNS < 20%. These malignancies represent 18% of the incident cancer cases, making up 11% and 22% for women and men, respectively [23]. 

For all malignancies combined, better survival was observed in women than in men, which is largely explained by the different distribution of cancer groups by sex, as has been mentioned above. Furthermore, women showed higher survival than did men for 12 out of 23 non-sex specific cancer groups. A better prognosis in women for several cancers has been consistently reported in other studies [24,25,26]. A biological advantage mediated by sexual hormones has been hypothesized [25,27]. Other considerations are that stage at diagnosis, tumor subsite and histology, and patients’ comorbidity may differ between the sexes [25,26]. Our study revealed a remarkable male survival disadvantage for oral cavity and pharynx cancer (absolute difference in 5y-ASNS point estimates of 19%), which could be partly related to differences in the prevalence of risk factors between sexes, such as smoking and alcohol consumption, that in turn are associated with histological type, subsite and comorbidity [25,28]. Nevertheless, the gender gap we observed for this cancer group warrants deeper examination, as it appears to be wider than described in other studies [28,29]. 

Survival generally decreased with age, consistent with other population-based studies [5,21,30,31]. Several factors may be contributing to age-related differences in survival, including comorbidity, frailty, socioeconomic factors, suboptimal cancer management, diagnostic delays, and patient’s preferences for treatment options [32,33,34]. Further studies are needed to better understand this age-related disparity and to guide strategies to reduce it.

Differences between observed and net survival were smaller for cancers that tend to occur among younger patients (e.g., testicle cancer or Hodgkin lymphoma) than for those cancers occurring in older patients (e.g., prostate cancer or chronic lymphoid leukemia), which is explained by less competing risks for death at a younger age. Likewise, as expected, these differences also tended to be smaller for cancers with poorer prognosis (e.g., pancreas or brain cancers). The large difference (>20 percentage points) between the 5y-ASNS and the conditional 5|1y-ASNS in patients with cancer of the esophagus, stomach and gallbladder possibly reflects the impact of postoperative mortality and/or comorbidity on first-year survival, as these patients frequently require high-risk procedures with a considerable incidence of major complications [35].

The CONCORD-3 study reported worldwide survival estimates for 15 cancer groups in adults, both sexes combined, up to the period 2010–2014 [4]. Spanish figures were within the ranges of most countries globally; however, the 5y-ASNS for esophageal, lung and brain cancer seem to be lower in Spain than in most high-income countries. Similarly, our 5y-ASNS estimates for esophageal and lung cancer tended to be lower than those of seven high-income countries published by the ICBP-SURVMARK2 project for 2010–2014 [31]. Different distribution by sex and smoking habits could be among the reasons for these differences, and variation in registration practices across countries may partly contribute. However, further investigation is required to determine whether potentially modifiable factors that could be intervened on, such as stage at diagnosis and access to timely and optimal treatment, are contributing to the poor prognosis for these tumors. A previous study found regional differences in survival for various cancers in Spain, most notably for lung cancer [36]. Indications that there is room for improvement have been seen in some high-resolution studies showing geographical variation in the early diagnosis and management of cancer patients in Spain [37,38]. Furthermore, some Spanish studies have reported geographical disparities in infrastructure and equipment for radiation oncology, and in access to oncology drugs and predictive biomarkers [39,40].

We observed significant increases in survival between periods of diagnosis, 2002–2007 and 2008–2013, for all cancers combined and for 19 of 29 cancer groups in both sexes (14 of 25 in men and 14 of 27 in women), and no significant decrease was found for any cancer group. For most cancers, larger improvements were found in patients younger than 75 years at diagnosis than in those aged 75 years and older, as described in other studies [31], and various factors might play a role in these differences, as mentioned previously.

Some of the largest increases were seen for some hematological malignancies: chronic myeloid leukemia, myeloma, non-Hodgkin lymphoma, acute myeloid leukemia, acute lymphoid leukemia and the group of leukemia NOS and others, although the increases were not statistically significant for the two latter groups. Chronic myeloid leukemia was the malignancy that showed the greatest increase in survival in the period 2000–2007 compared to 1995–1999 [7] and continued to rise considerably in 2008–2013. This results mostly from the introduction in 2001 of treatments with tyrosine kinase inhibitors, which dramatically changed the course and prognosis of the disease [41,42]. The increased survival in patients with myeloma is probably largely due to changes in the therapeutic arsenal in the last decade [43]. For non-Hodgkin lymphoma and some leukemias, advances in molecular biology and cytogenetic techniques have led to better diagnostic and therapeutic approaches that may be reflected in the observed improved survival [44,45]. Despite the aforementioned advances, no increased survival was observed for chronic lymphoid leukemia, but this may be at least partially due to a bias resulting from the change of diagnostic criteria in the 2008 WHO classification [46]. This change might have led to classification of some patients who formerly would have been diagnosed with chronic lymphoid leukemia, as monoclonal B-cell lymphocytosis, a premalignant condition [47].

Among the most common cancers, survival increases were particularly large for those of the colon and rectum, probably related to earlier diagnosis and better patient management [48]. Only a few regions of Spain had started population-based organized screening programs for colorectal cancer in the studied period, therefore, it is unlikely that there was already an impact on survival from these programs. An increase in survival was also observed for breast cancer, for which there are well-established screening programs in all Spanish regions. Regarding thyroid and kidney cancers, it should be note that some of the apparent improvement in survival might be caused by the increased diagnosis of indolent tumors [49,50,51]. 

Improved survival was also seen for some of the tumors with poor prognosis including pancreatic, esophageal, lung, liver and brain cancer, possibly due to advancements in diagnosis and treatment, together with enhanced supportive care [52,53]. However, we did not detect changes for gallbladder and stomach cancer, two other neoplasms that showed low survival. Moreover, for stomach cancer, the survival we found in the last period is very similar to that in 1995–1999 reported in a previous study [7]. The persisting poor prognosis for all these cancers underscores the need to strengthen primary prevention and early diagnosis whenever possible. 

The main strengths of this study include its population-based setting, which minimized the selection bias present in most clinical trials; the use of standardized registration procedures and high-quality data; and the reliable follow-up of vital status. Among the limitations, we acknowledge that our data covered only about 26% of the Spanish population, nevertheless this is the most complete and accurate information available on the survival of cancer patients in Spain. Another limitation is that some important explanatory factors, such as stage at diagnosis, treatments, or socioeconomic status, could not be considered because these data were not available. Finally, the fact that survival was estimated using the cohort approach for patients diagnosed in 2002–2007 and the period approach for those diagnosed in 2008–2013, might have slightly underestimated the level of survival improvement achieved [54]. 

## 5. Conclusions

We provided cancer survival estimates and analyzed survival trends among adult patients in Spain by cancer group, sex, and age. These survival estimates are crucial indicators for assessing the cancer strategy of the Spanish National Health System and the overall effectiveness of health services for cancer patients. We found survival improvements for most cancer groups and there may be multiple reasons for these findings, including earlier diagnosis and improved treatment options. However, the persistent poor prognosis observed for some cancers emphasizes the need to reinforce actions along the cancer continuum, from primary prevention to early diagnosis, optimal treatment, and supportive care. Further investigation of possible sociodemographic inequalities is warranted. Population-based cancer registries are fundamental to continue monitoring cancer survival.

## Figures and Tables

**Table 1:** Cancer groups, number of cases included and data quality indicators, 2002–2013.

Cancer Groups	ICD-10 Codes	Number of Eligible Cases	Excluded, %		Number of Cases Included (%)	Data Quality Indicators, %		
			DCO	Diagnosed at Autopsy		Microscopically Verified	Non-Specific Morphology ^a^	Lost to Follow-Up ^b^
Oral cavity and pharynx	C01–C06, C09–14	14,939	0.64	0.11	14,828 (99.26)	98.73	1.50	0.26
Esophagus	C15	6442	1.60	0.20	6326 (98.20)	95.78	4.47	0.40
Stomach	C16	21,748	2.21	0.43	21,175 (97.37)	94.63	5.77	0.25
Colon	C18	58,307	1.75	0.32	57,097 (97.92)	94.93	5.38	0.14
Rectum	C19–C20	28,990	0.63	0.20	28,750 (99.17)	97.26	3.01	0.15
Liver	C22	14,863	4.44	0.69	14,101 (94.87)	43.93	37.08	0.35
Gallbladder and bile ducts ^c^	C23–C24	6865	2.08	0.52	6686 (97.39)	65.99	34.89	0.27
Pancreas	C25	15,685	4.07	0.54	14,962 (95.39)	61.72	40.22	0.30
Larynx	C32	10,636	0.80	0.03	10,548 (99.17)	98.52	1.80	0.20
Lung, bronchus and trachea ^c^	C33–34	66,692	2.21	0.42	64,938 (97.37)	87.20	13.62	0.32
Skin melanoma	C43	12,857	0.32	0.05	12,809 (99.63)	99.63	0.00	0.30
Breast (women only)	C50	67,186	0.75	0.01	66,670 (99.23)	98.55	1.69	0.25
Cervix uteri	C53	5674	0.55	0.00	5643 (99.45)	98.90	1.45	0.87
Corpus uteri	C54	14,912	0.50	0.07	14,827 (99.43)	98.14	1.98	0.20
Ovary and annexes	C56, C570–C574, C577	8635	1.97	0.24	8444 (97.79)	91.38	9.53	0.28
Prostate	C61	77,920	1.40	0.19	76,682 (98.41)	91.38	8.62	0.16
Testicle	C62	3102	0.16	0.10	3094 (99.74)	99.22	0.94	0.52
Kidney	C64	13,912	1.32	0.71	13,630 (97.97)	83.09	17.23	0.21
Urinary bladder	C67, D090, D414	47,058	0.76	0.09	46,659 (99.15)	95.75	4.94	0.12
Brain	C71	9404	2.46	0.38	9137 (97.16)	69.94	18.44	0.42
Thyroid	C73	9176	0.20	0.73	9091 (99.07)	99.33	0.79	0.34
Hodgkin lymphoma	C81	3531	0.03	0.57	3510 (99.41)	99.97	0.00	0.34
Non-Hodgkin lymphoma	C82–C86, C96	18,691	0.81	0.56	18,435 (98.63)	98.06	9.74	0.23
Myeloma	C90	7421	1.68	0.19	7282 (98.13)	93.79	0.00	0.15
Acute lymphoid leukemia	C910	760	0.53	0.26	754 (99.21)	99.73	0.00	0.13
Chronic lymphoid leukemia	C911	5293	0.36	0.06	5271 (99.58)	99.37	0.00	0.23
Acute myeloid leukemia	C920, C923–C928, C930, C940–C946	4246	0.00	0.00	4246 (100.00)	99.48	0.00	0.33
Chronic myeloid leukemia	C921	1302	0.69	0.00	1293 (99.31)	99.85	0.00	0.23
Leukemia NOS and others	C913; C914; C915; C916; C917; C918; C919; C929; C931; C947; C950; C959	2321	11.72	0.26	2043 (88.02)	90.16	39.94	0.34
Other cancers ^d^		54,405	3.46	0.37	52,319 (96.17)	85.91	14.68	0.23
All cancers ^d^	C00–C96 (except C44), D090, D414, D45–D47	612,973	1.64	0.27	601,250 (98.09)	91.03	8.97	0.23

Abbreviations: ICD-10, International Classification of Diseases—10th revision; DCO, Cases known by death certificate only; NOS, not otherwise specified. ^a^ Non-specific morphology: International Classification of Diseases for Oncology codes, 3rd edition, 8000–8005 (solid tumors) and 9590, 9591, 9800, 9801, 9805, 9820, 9832 or 9860 (hematological neoplasms). ^b^ Cases censored alive before five years from diagnosis unless they were censored due to the end of follow-up (31 December 2015). ^c^ Note that the group referred in the text to as “gallbladder” includes gallbladder and bile ducts; and the group referred to as “lung” includes lung, bronchus and trachea. ^d^ Excluding non-melanoma skin cancer.

**Table 2:** Five-year observed survival (OS) and net survival (NS) by sex in adult patients diagnosed with cancer in Spain in 2008–2013.

Cancer Group		Men			Women			Both	
	Number of Cases	OS (95% CI), %	NS (95% CI), %	Number of Cases	OS (95% CI), %	NS (95% CI), %	Number of Cases	OS (95% CI), %	NS (95% CI), %
Oral cavity and pharynx	5573	37.2 (35.9–38.6)	40.5 (39.0–42.0)	1733	53.1 (50.5–55.5)	57.6 (54.7–60.6)	7306	41.0 (39.8–42.2)	44.5 (43.2–45.9)
Esophagus	2619	11.5 (10.2–12.9)	12.7 (11.3–14.2)	472	13.8 (10.7–17.3)	14.6 (11.0–18.1)	3091	11.9 (10.7–13.1)	13.0 (11.7–14.4)
Stomach	6533	20.8 (19.8–21.8)	24.0 (22.7–25.2)	3880	23.3 (21.9–24.7)	26.1 (24.5–27.7)	10,413	21.7 (20.9–22.6)	24.8 (23.8–25.8)
Colon	18,121	51.2 (50.4–52.0)	60.5 (59.5–61.5)	12,488	53.3 (52.3–54.2)	60.2 (59.1–61.3)	30,609	52.1 (51.5–52.7)	60.4 (59.7–61.1)
Rectum	9580	51.2 (50.1–52.3)	59.0 (57.7–60.4)	5136	53.9 (52.4–55.3)	59.7 (57.9–61.4)	14,716	52.1 (51.3–53.0)	59.3 (58.2–60.3)
Liver	5440	16.1 (15.1–17.2)	17.6 (16.4–18.8)	2028	10.3 (8.8–11.8)	11.2 (9.5–12.8)	7468	14.6 (13.7–15.5)	15.9 (14.9–16.9)
Gallbladder and bile ducts	1584	14.8 (12.9–16.7)	17.2 (14.9–19.5)	1766	12.0 (10.4–13.7)	14.2 (12.1–16.3)	3350	13.3 (12.1–14.6)	15.6 (14.1–17.2)
Pancreas	4427	5.7 (5.0–6.5)	6.2 (5.4–7.1)	3881	6.3 (5.5–7.2)	6.9 (5.9–7.8)	8308	6.0 (5.5–6.6)	6.5 (5.9–7.2)
Larynx	4460	55.2 (53.6–56.7)	61.5 (59.7–63.3)	355	64.7 (59.1–69.8)	68.0 (62.2–73.8)	4815	55.9 (54.3–57.3)	62.0 (60.3–63.7)
Lung	26,981	10.9 (10.5–11.3)	12.1 (11.6–12.6)	6352	17.3 (16.3–18.4)	18.0 (16.9–19.2)	33,333	12.1 (11.7–12.5)	13.2 (12.8–13.6)
Skin melanoma	3140	70.9 (69.2–72.6)	80.3 (78.1–82.5)	3480	81.4 (79.9–82.7)	87.5 (85.7–89.3)	6620	76.4 (75.3–77.5)	84.1 (82.7–85.5)
Breast (women)				34,294	82.0 (81.6–82.5)	87.3 (86.8–87.9)	34,294		
Cervix uteri				2726	65.8 (63.9–67.6)	67.6 (65.7–69.6)	2726		
Corpus uteri				7432	70.2 (69.1–71.3)	75.1 (73.8–76.4)	7432		
Ovary and annexes				4199	43.0 (41.3–44.6)	44.7 (43.0–46.4)	4199		
Prostate	38,929	78.0 (77.5–78.4)	90.0 (89.4–90.6)				38,929		
Testicle	1671	95.1 (93.9–96.1)	95.9 (94.8–97.0)				1671		
Kidney	5069	58.5 (57.0–59.9)	65.4 (63.6–67.2)	2374	59.6 (57.4–61.6)	64.1 (61.7–66.5)	7443	58.8 (57.6–60.0)	65.0 (63.5–66.4)
Urinary bladder	20,022	60.3 (59.5–61.0)	70.8 (69.9–71.8)	3973	61.6 (60.0–63.2)	70.7 (68.6–72.8)	23,995	60.5 (59.8–61.1)	70.8 (69.9–71.7)
Brain	2613	13.5 (12.2–14.9)	13.9 (12.5–15.4)	2104	14.0 (12.4–15.6)	14.3 (12.7–15.9)	4717	13.7 (12.7–14.8)	14.1 (13.0–15.2)
Thyroid	1186	84.8 (82.5–86.9)	88.9 (86.5–91.4)	4151	93.0 (92.1–93.7)	95.3 (94.5–96.2)	5337	91.2 (90.4–92.0)	93.9 (93.1–94.8)
Hodgkin lymphoma	1026	78.5 (75.8–81.0)	81.1 (78.3–83.8)	725	82.4 (79.4–85.1)	83.5 (80.6–86.5)	1751	80.2 (78.1–82.0)	82.1 (80.0–84.1)
Non-Hodgkin lymphoma	5188	57.6 (56.1–59.0)	63.8 (62.1–65.4)	4395	62.2 (60.7–63.7)	66.7 (64.9–68.4)	9583	59.7 (58.6–60.7)	65.1 (63.9–66.3)
Myeloma	1989	35.1 (32.8–37.4)	40.2 (37.4–43.0)	1747	41.2 (38.7–43.7)	45.0 (42.2–47.9)	3736	38.0 (36.3–39.7)	42.5 (40.5–44.5)
Acute lymphoid leukemia	189	36.5 (29.5–43.6)	38.0 (30.6–45.4)	147	32.1 (24.5–39.9)	32.7 (24.6–40.7)	336	34.6 (29.4–39.8)	35.7 (30.2–41.1)
Chronic lymphoid leukemia	1446	61.4 (58.6–64.0)	73.1 (69.4–76.9)	995	64.1 (60.8–67.1)	74.2 (70.1–78.2)	2441	62.5 (60.4–64.5)	73.6 (70.8–76.3)
Acute myeloid leukemia	1192	19.3 (17.0–21.7)	20.1 (17.6–22.6)	1022	25.9 (23.1–28.7)	26.3 (23.3–29.3)	2214	22.3 (20.5–24.2)	22.9 (21.0–24.9)
Chronic myeloid leukemia	366	69.9 (64.7–74.4)	75.1 (69.5–80.8)	240	70.3 (63.7–75.9)	74.2 (67.0–81.3)	606	70.0 (66.0–73.7)	74.8 (70.4–79.2)
Leukemia NOS and others	658	32.8 (28.9–36.8)	37.8 (32.5–43.1)	405	30.0 (25.1–35.0)	33.2 (27.3–39.1)	1063	31.7 (28.6–34.8)	36.0 (32.0–40.0)
All cancers ^a^	184,991	47.4 (47.1–47.6)	54.3 (54.0–54.6)	123,769	57.4 (57.1–57.7)	62.0 (61.6–62.3)	308,760	51.4 (51.2–51.6)	57.4 (57.2–57.6)

Survival estimates are non-age standardized. Abbreviations: NOS, not otherwise specified. ^a^ Excluding non-melanoma skin cancer.

**Table 3:** Age-standardised net survival at one, three and five years post-diagnosis and at five years post-diagnosis conditional on having survived one year, in adult patients diagnosed with cancer in Spain in 2008–2013.

Cancer Groups	Age-Standardised Net Survival (95% CI), %			
	1 Year	3 Years	5 Years	5 Years Conditional
Oral cavity and pharynx	70.2 (69.1–71.4)	51.0 (49.7–52.3)	42.9 (41.5–44.4)	60.7 (58.7–62.7)
Esophagus	38.1 (36.3–39.9)	17.4 (16.0–19.0)	13.5 (12.1–15.0)	34.3 (31.0–37.9)
Stomach	51.2 (50.2–52.2)	31.7 (30.7–32.7)	27.4 (26.4–28.4)	52.8 (51.1–54.6)
Colon	81.3 (80.8–81.7)	69.1 (68.5–69.7)	63.3 (62.6–64.0)	77.6 (76.9–78.4)
Rectum	83.4 (82.8–84.0)	68.7 (67.9–69.5)	61.1 (60.1–62.1)	72.8 (71.7–73.9)
Liver	45.8 (44.6–47.0)	25.1 (24.0–26.2)	17.4 (16.4–18.5)	36.0 (34.0–38.0)
Gallbladder and bile ducts	45.0 (42.9–47.2)	27.3 (25.3–29.4)	20.9 (18.9–23.1)	44.3 (40.9–48.0)
Pancreas	27.9 (26.9–29.0)	11.5 (10.7–12.3)	8.3 (7.5–9.1)	26.8 (24.4–29.4)
Larynx	84.1 (82.9–85.3)	68.1 (66.5–69.8)	60.5 (58.6–62.5)	71.6 (69.5–73.9)
Lung	39.0 (38.5–39.6)	18.6 (18.2–19.1)	13.8 (13.3–14.2)	33.7 (32.6–34.7)
Skin melanoma	95.6 (95.1–96.2)	89.4 (88.5–90.2)	85.8 (84.7–86.9)	89.6 (88.6–90.7)
Breast (women)	96.0 (95.7–96.3)	90.1 (89.6–90.5)	85.5 (84.8–86.2)	88.8 (88.1–89.5)
Cervix uteri	84.3 (82.8–85.7)	70.7 (68.9–72.6)	65.5 (63.5–67.5)	76.6 (74.3–78.9)
Corpus uteri	89.2 (88.4–89.9)	78.5 (77.5–79.6)	74.0 (72.8–75.3)	82.3 (80.9–83.7)
Ovary and annexes	70.4 (69.1–71.8)	50.8 (49.2–52.4)	40.9 (39.3–42.6)	55.7 (53.3–58.1)
Prostate	96.4 (96.1–96.6)	92.2 (91.7–92.6)	89.6 (89.0–90.1)	92.3 (91.7–92.9)
Testicle	93.2 (90.6–95.9)	91.5 (88.2–95.0)	89.4 (85.2–93.9)	95.3 (90.6–100.0)
Kidney	78.9 (78.0–79.9)	70.2 (69.0–71.3)	65.0 (63.6–66.4)	81.6 (80.0–83.2)
Urinary bladder	87.9 (87.5–88.4)	78.6 (78.0–79.2)	74.1 (73.3–74.8)	83.7 (83.0–84.5)
Brain	48.9 (47.5–50.2)	26.8 (25.5–28.2)	22.2 (20.8–23.6)	36.2 (33.7–38.8)
Thyroid	93.9 (93.1–94.7)	92.3 (91.3–93.2)	91.4 (90.2–92.6)	97.1 (95.9–98.3)
Hodgkin lymphoma	89.4 (88.1–90.8)	83.1 (81.4–84.8)	81.2 (79.3–83.1)	89.5 (87.3–91.8)
Non-Hodgkin lymphoma	78.1 (77.2–78.9)	68.9 (67.9–69.9)	65.0 (63.8–66.2)	82.2 (80.9–83.6)
Myeloma	79.5 (78.2–80.8)	60.4 (58.7–62.1)	47.8 (45.9–49.8)	58.6 (56.4–60.9)
Acute lymphoid leukemia	64.1 (59.3–69.2)	43.7 (38.5–49.5)	40.6 (35.4–46.7)	61.9 (54.8–69.9)
Chronic lymphoid leukemia	87.2 (86.2–88.2)	86.7 (85.2–88.3)	78.9 (76.7–81.1)	83.2 (80.9–85.5)
Acute myeloid leukemia	42.9 (40.9–45.1)	25.1 (23.3–27.1)	21.7 (19.9–23.7)	40.4 (36.6–44.6)
Chronic myeloid leukemia	86.4 (83.4–89.5)	76.9 (73.0–81.0)	70.8 (66.2–75.6)	79.6 (74.4–85.2)
Leukemia NOS and others	68.5 (65.5–71.8)	51.4 (47.9–55.1)	44.3 (40.6–48.3)	61.9 (57.4–66.9)
All cancers ^a^	74.7 (74.6–74.9)	63.0 (62.8–63.2)	58.2 (58.0–58.4)	77.1 (76.8–77.3)

Abbreviations: NOS, not otherwise specified. ^a^ Excluding non-melanoma skin cancer.

**Table 4:** Five-year age-standardised net survival (ASNS) in men and women diagnosed with cancer in Spain in 2002–2007 and 2008–2013 and absolute change between periods.

Cancer Group	Men			Women		
	ASNS (95% CI), %		Absolute Change ^a^ (95% CI), %	ASNS (95% CI), %		Absolute Change ^a^ (95% CI), %
	2002–2007	2008–2013		2002–2007	2008–2013	
Oral cavity and pharynx	37.4 (35.7, 39.1)	38.2 (36.6, 39.9)	0.9 (−1.5, 3.2)	51.6 (48.7, 54.6)	57.2 (54.4, 60.2)	**5.6 (1.5, 9.8)**
Esophagus	9.7 (8.6, 11.0)	13.1 (11.6, 14.7)	**3.4 (1.4, 5.3)**	17.3 (13.8, 21.6)	16.5 (13.1, 20.8)	−0.8 (−6.2, 4.6)
Stomach	24.6 (23.5, 25.8)	26.0 (24.8, 27.4)	1.4 (−0.3, 3.2)	30.6 (29.0, 32.3)	30.3 (28.6, 32.2)	−0.3 (−2.8, 2.2)
Colon	57.5 (56.5, 58.4)	63.1 (62.2, 64.1)	**5.6 (4.3, 7.0)**	59.8 (58.7, 60.8)	63.9 (62.8, 64.9)	**4.1 (2.6, 5.6)**
Rectum	55.8 (54.6, 57.1)	60.4 (59.1, 61.7)	**4.6 (2.8, 6.4)**	58.1 (56.6, 59.7)	62.7 (61.1, 64.3)	**4.6 (2.3, 6.8)**
Liver	15.2 (14.1, 16.3)	17.9 (16.7, 19.2)	**2.8 (1.1, 4.4)**	16.4 (14.3, 18.7)	16.2 (14.1, 18.6)	−0.2 (−3.4, 2.9)
Gallbladder and bile ducts	18.7 (16.2, 21.6)	22.3 (19.6, 25.3)	3.6 (−0.3, 7.5)	18.1 (15.7, 20.8)	19.2 (16.5, 22.4)	1.1 (−2.8, 5.0)
Pancreas	5.7 (4.9, 6.6)	7.2 (6.3, 8.2)	**1.5 (0.2, 2.8)**	7.3 (6.2, 8.5)	10.0 (8.7, 11.4)	**2.7 (1.0, 4.5)**
Larynx	60.6 (58.8, 62.4)	60.0 (58.0, 62.1)	−0.5 (−3.2, 2.2)	68.5 (62.4, 75.1)	66.1 (58.8, 74.2)	−2.4 (−12.4, 7.5)
Lung	11.2 (10.7, 11.6)	12.7 (12.2, 13.2)	**1.6 (0.9, 2.2)**	16.2 (15.1, 17.4)	17.6 (16.5, 18.8)	1.4 (−0.3, 3.0)
Skin melanoma	79.1 (77.3, 81.0)	82.3 (80.5, 84.1)	**3.2 (0.6, 5.7)**	88.6 (87.2, 90.0)	88.9 (87.5, 90.3)	0.3 (−1.7, 2.3)
Breast (women)				83.2 (82.5, 83.9)	85.5 (84.8, 86.2)	**2.3 (1.3, 3.3)**
Cervix uteri				64.4 (62.5, 66.3)	65.5 (63.5, 67.5)	1.1 (−1.7, 3.8)
Corpus uteri				74.6 (73.4, 75.9)	74.0 (72.8, 75.3)	−0.6 (−2.4, 1.1)
Ovary and annexes				37.5 (36.0, 39.0)	40.9 (39.3, 42.6)	**3.5 (1.2, 5.7)**
Prostate	87.8 (87.2, 88.4)	89.6 (89.0, 90.1)	**1.7 (0.9, 2.6)**			
Testicle	85.2 (81.0, 89.6)	89.4 (85.2, 93.9)	4.2 (−1.9, 10.3)			
Kidney	59.8 (58.1, 61.6)	64.8 (63.0, 66.6)	**5.0 (2.4, 7.5)**	61.4 (59.1, 63.7)	65.8 (63.7, 68.0)	**4.4 (1.3, 7.6)**
Urinary bladder	72.3 (71.5, 73.1)	73.8 (73.0, 74.7)	**1.6 (0.4, 2.7)**	72.9 (71.1, 74.8)	75.9 (74.2, 77.6)	**3.0 (0.5, 5.5)**
Brain	19.3 (17.8, 21.0)	20.8 (19.2, 22.6)	1.5 (−0.9, 3.9)	21.2 (19.3, 23.2)	24.2 (22.1, 26.5)	**3.1 (0.2, 6.0)**
Thyroid	78.3 (74.9, 81.8)	86.1 (83.2, 89.1)	**7.8 (3.3, 12.3)**	88.8 (87.3, 90.3)	93.1 (91.8, 94.4)	**4.3 (2.3, 6.3)**
Hodgkin lymphoma	80.0 (77.3, 82.7)	80.6 (77.8, 83.4)	0.6 (−3.3, 4.5)	83.3 (80.8, 85.9)	82.6 (80.0, 85.3)	−0.7 (−4.4, 3.0)
Non-Hodgkin lymphoma	57.2 (55.4, 58.9)	62.4 (60.7, 64.1)	**5.2 (2.8, 7.6)**	63.0 (61.4, 64.6)	68.4 (66.8, 70.0)	**5.4 (3.2, 7.7)**
Myeloma	40.1 (37.7, 42.7)	44.8 (42.2, 47.5)	**4.6 (1.0, 8.3)**	42.5 (40.0, 45.2)	51.2 (48.5, 54.1)	**8.7 (4.9, 12.5)**
Acute lymphoid leukemia	35.1 (29.9, 41.3)	41.6 (34.8, 49.6)	6.5 (−2.9, 15.8)	37.1 (30.4, 45.3)	40.0 (32.1, 49.7)	2.9 (−8.5, 14.3)
Chronic lymphoid leukemia	78.8 (76.2, 81.5)	77.7 (74.6, 80.8)	−1.1 (−5.2, 2.9)	80.7 (77.7, 83.9)	80.7 (77.7, 83.8)	−0.0 (−4.4, 4.3)
Acute myeloid leukemia	17.0 (14.9, 19.4)	19.2 (16.9, 21.8)	2.2 (−1.1, 5.5)	19.8 (17.3, 22.6)	24.9 (22.1, 28.0)	**5.1 (1.2, 9.0)**
Chronic myeloid leukemia	59.2 (53.6, 65.3)	68.8 (62.7, 75.4)	**9.6 (1.0, 18.2)**	67.5 (59.6, 76.5)	73.0 (66.4, 80.3)	5.5 (−5.4, 16.4)
Leukemia NOS and others	42.5 (38.2, 47.2)	45.9 (41.4, 50.9)	3.4 (−3.1, 10.0)	33.5 (28.5, 39.5)	41.9 (36.1, 48.7)	**8.4 (0.0, 16.8)**
All cancers ^b^	52.0 (51.7, 52.2)	55.3 (55.0, 55.6)	**3.3 (3.0, 3.7)**	59.2 (58.9, 59.6)	61.7 (61.4, 62.1)	**2.5 (2.0, 3.0)**

Abbreviations: NOS, not otherwise specified. ^a^ Absolute difference = ASNS 2nd period—ASNS 1st period. Bold identifies statistically significant changes. ^b^ Excluding non-melanoma skin cancer.

**Table 5:** Five-year age-standardised net survival (ASNS) in patients diagnosed with cancer in Spain in 2002–2007 and 2008–2013 and absolute change between periods according to age group at diagnosis.

Cancer Group	Younger Than 75 Years			Aged 75 Years and Older		
	ASNS (95% CI), %		Absolute Change ^a^ (95% CI), %	ASNS (95% CI), %		Absolute Change ^a^ (95% CI), %
	2002–2007	2008–2013		2002–2007	2008–2013	
Oral cavity and pharynx	41.6 (40.3, 42.9)	46.6 (45.2, 48.2)	**5.1 (3.1, 7.1)**	35.9 (32.1, 39.6)	33.8 (30.3–37.3)	−2.1 (−7.2, 3.1)
Esophagus	12.8 (11.4, 14.3)	16.2 (14.4, 18.1)	**3.4 (1.1, 5.7)**	4.5 (2.7, 6.2)	7.0 (4.8–9.2)	2.5 (−0.2, 5.3)
Stomach	30.5 (29.3, 31.8)	31.5 (30.2, 32.9)	1.0 (−0.8, 2.8)	17.2 (15.7, 18.6)	17.3 (15.8–18.7)	0.1 (−1.9, 2.1)
Colon	63.0 (62.1, 63.8)	68.9 (68.1, 69.8)	**6.0 (4.8, 7.1)**	47.1 (45.7, 48.5)	49.7 (48.4–51.0)	**2.5 (0.6, 4.4)**
Rectum	61.8 (60.8, 62.9)	67.3 (66.2, 68.5)	**5.5 (3.9, 7.1)**	43.7 (41.7, 45.7)	45.9 (43.9–48.0)	2.3 (−0.6, 5.1)
Liver	18.8 (17.6, 20.1)	21.6 (20.2, 23.0)	**2.8 (0.9, 4.6)**	6.3 (5.1, 7.6)	7.1 (5.8–8.4)	0.8 (−1.0, 2.6)
Gallbladder and bile ducts	21.5 (19.2, 24.1)	25.2 (22.6, 28.2)	**3.7 (0.0, 7.4)**	10.6 (8.4, 12.8)	10.3 (8.4–12.3)	−0.3 (−3.2, 2.7)
Pancreas	7.9 (7.0, 8.8)	10.7 (9.7, 11.8)	**2.8 (1.4, 4.2)**	2.0 (1.3, 2.7)	2.3 (1.6–3.0)	0.3 (−0.7, 1.3)
Larynx	64.5 (63.0, 66.1)	65.2 (63.3, 67.0)	0.7 (−1.8, 3.1)	51.5 (47.0, 56.0)	49.1 (44.1–54.0)	−2.4 (−9.1, 4.3)
Lung	14.2 (13.8, 14.7)	17.0 (16.4, 17.6)	**2.7 (2.0, 3.5)**	5.9 (5.3, 6.5)	5.9 (5.3–6.6)	0.0 (−0.8, 0.9)
Skin melanoma	86.3 (85.2, 87.3)	87.8 (86.7, 88.9)	**1.5 (0.0, 3.1)**	71.3 (66.5, 76.2)	73.6 (69.2–78.0)	2.2 (−4.3, 8.8)
Breast (women)	87.5 (87.0, 88.1)	90.6 (90.1, 91.1)	**3.1 (2.3, 3.8)**	72.5 (70.4, 74.7)	72.8 (70.8–74.9)	0.3 (−2.6, 3.3)
Cervix uteri	69.6 (67.6, 71.5)	70.2 (68.1, 72.3)	0.6 (−2.3, 3.5)	32.8 (26.6, 39.0)	36.5 (30.1–42.9)	3.7 (−5.2, 12.6)
Corpus uteri	81.3 (80.2, 82.4)	82.3 (81.1, 83.5)	1.0 (−0.6, 2.6)	58.4 (55.3, 61.5)	53.8 (50.5–57.1)	−4.6 (−9.1, 0.0)
Ovary and annexes	46.1 (44.3, 48.0)	49.8 (47.8, 51.9)	**3.7 (1.0, 6.5)**	16.3 (13.5, 19.1)	19.1 (16.1–22.0)	2.8 (−1.3, 6.9)
Prostate	92.5 (92.0, 93.1)	95.6 (95.1, 96.1)	**3.1 (2.3, 3.9)**	76.3 (74.8, 77.9)	74.7 (73.2, 76.3)	−1.6 (−3.8, 0.6)
Testicle	91.3 (88.2, 94.6)	93.0 (89.6, 96.6)	1.7 (−3.1, 6.4)	29.9 (0.0, 62.0)	57.0 (27.4–86.7)	27.1 (−16.6, 70.7)
Kidney	67.3 (65.8, 68.8)	72.7 (71.3, 74.2)	**5.5 (3.4, 7.6)**	42.5 (39.4, 45.7)	46.0 (42.8–49.2)	3.5 (−1.1, 8.0)
Urinary bladder	78.6 (77.8, 79.4)	80.9 (80.1, 81.7)	**2.3 (1.2, 3.4)**	56.7 (55.0, 58.4)	57.3 (55.7–59.0)	0.7 (−1.7, 3.1)
Brain	22.8 (21.4, 24.2)	25.4 (23.9, 27.0)	**2.6 (0.5, 4.7)**	3.5 (2.2, 4.8)	2.4 (1.3–3.4)	−1.1 (−2.8, 0.5)
Thyroid	92.1 (91.0, 93.3)	95.4 (94.5, 96.3)	**3.3 (1.8, 4.8)**	50.3 (43.1, 57.6)	67.0 (60.5–73.4)	**16.6 (6.9, 26.3)**
Hodgkin lymphoma	85.8 (84.1, 87.6)	86.2 (84.4, 88.1)	0.4 (−2.2, 2.9)	40.3 (30.2, 50.3)	36.0 (26.1–45.9)	−4.2 (−18.4, 9.9)
Non-Hodgkin lymphoma	68.0 (66.7, 69.3)	73.8 (72.5, 75.1)	**5.8 (4.0, 7.7)**	39.2 (36.7, 41.8)	43.5 (40.9–46.1)	**4.3 (0.6, 7.9)**
Myeloma	49.1 (46.8, 51.4)	56.3 (53.9, 58.8)	**7.2 (3.9, 10.6)**	22.2 (19.5, 24.9)	26.9 (23.9–30.0)	**4.7 (0.7, 8.8)**
Acute lymphoid leukemia	39.3 (34.6, 44.7)	43.5 (37.8, 50.1)	4.2 (−3.8, 12.1)	2.6 (0.0, 6.6)	14.8 (4.4–25.1)	**12.2 (1.1, 23.3)**
Chronic lymphoid leukemia	85.5 (83.5, 87.6)	86.4 (84.0, 88.9)	0.9 (−2.2, 4.0)	64.9 (59.8, 70.0)	60.4 (55.5–65.3)	−4.5 (−11.6, 2.6)
Acute myeloid leukemia	24.3 (22.1, 26.8)	29.8 (27.3, 32.5)	**5.4 (1.9, 8.9)**	3.2 (1.6, 4.8)	2.1 (0.7–3.4)	−1.2 (−3.3, 0.9)
Chronic myeloid leukemia	75.0 (70.1, 80.1)	83.7 (78.9, 88.9)	**8.8 (1.7, 15.8)**	31.6 (18.7, 44.5)	39.0 (28.3–49.7)	7.4 (−9.4, 24.2)
Leukemia NOS and others	47.5 (43.2, 52.3)	53.9 (49.2, 59.1)	6.4 (−0.3, 13.1)	18.7 (14.4, 23.0)	20.7 (15.4–26.1)	2.0 (−4.9, 8.9)
All cancers ^b^	60.6 (60.4, 60.8)	65.1 (64.8, 65.3)	**4.5 (4.1, 4.8)**	41.5 (41.0, 42.0)	41.4 (40.9–41.8)	−0.1 (−0.8, 0.5)

Abbreviations: NOS, not otherwise specified. ^a^ Absolute difference = ASNS 2nd period—ASNS 1st period. Bold identifies statistically significant changes. ^b^ Excluding non-melanoma skin cancer.

## Data Availability

Data are available from the authors on reasonable request with the permission of the cancer registries. Data requestors will need to sign a data access agreement.

## Supplementary Materials

The following supporting information can be downloaded at: https://www.mdpi.com/article/10.3390/cancers14102441/s1, Table S1: Participating cancer registries, number of cases and periods of diagnosis included; Table S2: Five-year net survival by age group and sex for adult patients diagnosed with cancer in Spain in 2008–2013.
