# Supplementary material for: Cancer Survival in Adults in Spain: A Population-Based Study of the Spanish Network of Cancer Registries (REDECAN)
Source: Cancers (Basel). 2022 May 15;14(10):2441. doi: 10.3390/cancers14102441 (PMC9139549; doi:10.3390/cancers14102441)
Supplement: Supplementary file 1 [file cancers-14-02441-s001.zip › cancers-1729823-supplementary.pdf]

Supplementary Material

Supplement to: Guevara, M.; Molinuevo A.; Salmerón, D.; Marcos-Gragera, R.; Carulla, M.; Chirlaque, M.-D.; Rodríguez Camblor, M.; Alemán, A.; Rojas, D.; Vizcaíno Batllés, A.; et al. Cancer Survival in Adults in Spain: A Population-Based Study of the Spanish Network of Cancer Registries (REDECAN). *Cancers* 2022.

Supplementary Table S1. Participating cancer registries, number of cases and periods of diagnosis included.

Cancer registry	N	First period	Last period	End of follow-up
Asturias CR	54,417	2002–2007	2008–2010	2015
Canary Islands CR	78,733	2002–2007	2008–2013	2015
Castellón CR	26,018			
Breast cancer		2002–2007	2008–2013	2015
All other cancers		2004–2007	2008–2013	2015
Ciudad Real CR	18,481	2004–2007	2004–2011	2015
Cuenca CR	10,161	2002–2007	2008–2011	2015
Basque Country CR	150,328	2002–2007	2008–2013	2015
Girona CR	39,695	2002–2007	2008–2013	2015
Granada CR	43,625	2002–2007	2008–2013	2015
La Rioja CR	19,099	2002–2007	2008–2013	2015
Mallorca CR	35,366	2003–2007	2008–2012	2015
Murcia CR	48,252	2002–2007	2008–2010	2015
Navarra CR	34,357	2002–2007	2008–2012	2015
Tarragona CR	42,718	2002–2007	2008–2013	2015

Abbreviations: CR, cancer registry.

Supplementary Table S2. Five-year net survival by age group and sex for adult patients diagnosed with cancer in Spain in 2008–2013.

Cancer group	sex	Five-year net survival (95% CI), %				
		Age group				
		15-44 y	45-54 y	55-64 y	65-74 y	≥75 y
Oral cavity and pharynx						
	Men	62.5 (56.7–68.4)	47.2 (44.0–50.3)	42.4 (39.9–44.9)	36.0 (32.9–39.0)	27.6 (23.6–31.6)
	Women	81.4 (74.6–88.2)	67.7 (62.2–73.3)	61.0 (55.7–66.3)	57.2 (51.2–63.2)	44.0 (37.7–50.4)
	Both	68.3 (63.7–72.9)	51.6 (48.8–54.3)	45.6 (43.3–47.9)	40.2 (37.4–42.9)	33.8 (30.3–37.3)
Esophagus						
	Men	25.9 (14.7–37.0)	16.3 (12.2–20.4)	13.4 (10.8–16.1)	14.3 (11.5–17.2)	7.1 (4.6–9.5)
	Women	35.9 (12.3–59.4)	15.3 (6.2–24.4)	22.2 (14.0–30.5)	17.5 (9.3–25.6)	6.8 (2.5–11.1)
	Both	27.7 (17.4–38.0)	16.2 (12.4–19.9)	14.5 (12.0–17.1)	14.7 (12.0–17.3)	7.0 (4.8–9.2)
Stomach						
	Men	37.4 (30.6–44.2)	33.6 (29.5–37.6)	30.6 (27.8–33.4)	26.4 (24.0–28.7)	16.2 (14.2–18.1)
	Women	34.0 (26.6–41.4)	37.6 (31.9–43.2)	37.0 (32.4–41.7)	32.8 (29.2–36.4)	18.6 (16.5–20.8)
	Both	35.9 (30.8–40.9)	35.0 (31.7–38.3)	32.4 (30.0–34.8)	28.3 (26.4–30.3)	17.3 (15.8–18.7)
Colon						
	Men	72.3 (67.5–77.1)	70.3 (67.5–73.1)	70.5 (68.8–72.2)	65.4 (63.8–67.0)	49.8 (48.0–51.6)
	Women	69.9 (65.1–74.7)	68.9 (65.8–71.9)	72.4 (70.2–74.5)	68.0 (66.0–69.9)	49.5 (47.6–51.3)
	Both	71.1 (67.7–74.5)	69.6 (67.6–71.7)	71.2 (69.8–72.5)	66.3 (65.1–67.6)	49.7 (48.4–51.0)
Rectum						
	Men	70.5 (64.5–76.5)	68.7 (65.3–72.1)	68.5 (66.2–70.7)	62.2 (59.9–64.4)	46.3 (43.6–48.9)
	Women	72.0 (64.9–79.0)	72.9 (68.9–76.8)	71.8 (68.7–74.9)	66.2 (63.2–69.2)	45.4 (42.4–48.5)
	Both	71.1 (66.5–75.7)	70.4 (67.8–72.9)	69.5 (67.7–71.3)	63.4 (61.6–65.2)	45.9 (43.9–48.0)
Liver						
	Men	29.5 (21.7–37.3)	26.4 (23.2–29.7)	24.3 (21.6–26.9)	16.4 (14.1–18.6)	8.1 (6.3–10.0)
	Women	39.5 (23.3–55.7)	22.3 (14.1–30.5)	24.4 (18.6–30.1)	12.1 (8.9–15.4)	5.6 (3.8–7.3)
	Both	31.5 (24.4–38.6)	25.9 (22.9–29.0)	24.2 (21.8–26.6)	15.3 (13.4–17.2)	7.1 (5.8–8.4)

Gallbladder and bile ducts						
	Men	49.5 (28.8–70.2)	33.8 (23.2–44.4)	25.2 (19.1–31.2)	20.5 (16.1–24.8)	10.4 (7.5–13.3)
	Women	43.5 (22.0–65.0)	23.7 (11.8–35.6)	21.7 (15.5–27.8)	18.4 (13.8–22.9)	10.3 (7.7–12.9)
	Both	46.0 (30.5–61.5)	30.0 (22.0–37.9)	23.5 (19.2–27.9)	19.5 (16.4–22.7)	10.3 (8.4–12.3)
Pancreas						
	Men	21.8 (14.1–29.5)	10.9 (7.8–14.1)	9.5 (7.4–11.6)	5.5 (4.0–7.0)	1.9 (0.9–3.0)
	Women	25.9 (17.0–34.7)	17.1 (12.1–22.2)	13.5 (10.3–16.8)	7.8 (5.8–9.7)	2.5 (1.6–3.4)
	Both	23.7 (17.9–29.5)	13.1 (10.4–15.8)	10.9 (9.1–12.7)	6.4 (5.2–7.6)	2.3 (1.6–3.0)
Larynx						
	Men	71.0 (62.3–79.7)	65.4 (61.7–69.0)	67.0 (64.2–69.8)	60.8 (57.4–64.2)	48.9 (43.8–54.0)
	Women	85.5 (70.3–100.7)	69.3 (59.2–79.3)	65.6 (55.7–75.4)	74.1 (62.6–85.7)	52.4 (30.7–74.0)
	Both	73.3 (65.5–81.1)	65.8 (62.4–69.3)	66.9 (64.3–69.6)	61.5 (58.3–64.8)	49.1 (44.1–54.0)
Lung						
	Men	21.0 (17.2–24.7)	15.4 (13.9–16.8)	16.0 (15.0–17.0)	14.2 (13.3–15.1)	5.5 (4.9–6.2)
	Women	29.1 (24.0–34.1)	21.5 (19.2–23.9)	22.2 (20.0–24.4)	19.0 (16.6–21.5)	8.1 (6.1–10.0)
	Both	24.4 (21.3–27.5)	17.4 (16.1–18.6)	17.2 (16.3–18.1)	14.9 (14.0–15.7)	5.9 (5.3–6.6)
Skin melanoma						
	Men	88.7 (86.0–91.3)	85.3 (81.6–89.1)	82.6 (78.9–86.3)	78.2 (74.0–82.5)	71.1 (64.7–77.5)
	Women	95.8 (94.3–97.3)	91.3 (88.8–93.8)	90.4 (87.7–93.1)	84.7 (81.0–88.4)	76.1 (70.0–82.1)
	Both	92.8 (91.3–94.2)	88.7 (86.6–90.9)	86.6 (84.3–88.9)	81.3 (78.4–84.1)	73.6 (69.2–78.0)
Breast						
	Women	90.5 (89.6–91.4)	92.6 (92.0–93.3)	92.5 (91.7–93.2)	88.3 (87.2–89.4)	72.8 (70.8–74.9)
Cervix uteri						
	Women	81.0 (78.2–83.8)	74.2 (70.8–77.6)	67.0 (62.4–71.7)	54.8 (48.8–60.8)	36.5 (30.1–42.9)
Corpus uteri						
	Women	87.6 (83.5–91.6)	89.2 (87.2–91.2)	85.0 (83.3–86.7)	76.0 (73.7–78.2)	53.8 (50.5–57.1)
Ovary and annexes						
	Women	71.2 (66.9–75.5)	63.0 (59.3–66.6)	51.2 (47.6–54.8)	38.2 (34.5–41.9)	19.1 (16.1–22.0)
Prostate[a]		15-54 y	55-64 y	65-74 y	75-84 y	≥85 y
	Men	95.2 (93.9–96.5)	96.4 (95.8–97.1)	95.3 (94.6–96.0)	83.6 (82.1–85.0)	40.8 (36.0–45.6)

		Age group				
		15-44 y	45-54 y	55-64 y	65-74 y	≥75 y
Testicle						
	Men	97.2 (96.2–98.1)	92.1 (86.3–97.9)	96.8 (86.2–107.5)	65.4 (36.6–94.2)	57.0 (27.4–86.7)
Kidney						
	Men	80.0 (75.6–84.5)	76.7 (73.3–80.1)	72.7 (69.8–75.7)	66.9 (63.7–70.2)	47.7 (43.4–52.1)
	Women	90.1 (85.4–94.8)	78.4 (73.3–83.6)	74.7 (70.4–79.0)	70.2 (65.9–74.5)	43.2 (38.6–47.8)
	Both	83.5 (80.1–86.8)	77.2 (74.4–80.0)	73.3 (70.9–75.7)	67.9 (65.3–70.5)	46.0 (42.8–49.2)
Urinary bladder						
	Men	90.7 (87.9–93.5)	88.2 (86.4–90.0)	80.5 (79.1–81.9)	74.9 (73.4–76.3)	57.5 (55.6–59.4)
	Women	89.8 (84.4–95.2)	87.0 (83.6–90.4)	87.7 (84.8–90.6)	77.9 (74.6–81.2)	56.6 (52.8–60.3)
	Both	90.5 (88.0–93.0)	88.0 (86.4–89.5)	81.5 (80.2–82.8)	75.3 (73.9–76.6)	57.3 (55.7–59.0)
Brain						
	Men	54.3 (49.1–59.6)	18.7 (14.8–22.7)	6.7 (4.5–8.9)	3.8 (2.0–5.6)	2.0 (0.7–3.3)
	Women	57.8 (51.4–64.2)	27.1 (21.3–32.9)	11.0 (7.8–14.2)	3.8 (2.0–5.6)	2.7 (1.2–4.2)
	Both	55.8 (51.7–59.8)	21.8 (18.5–25.1)	8.5 (6.6–10.3)	3.7 (2.4–5.0)	2.4 (1.3–3.4)
Thyroid						
	Men	97.5 (95.7–99.4)	93.5 (89.5–97.5)	93.7 (89.5–97.9)	77.1 (68.7–85.4)	55.8 (40.9–70.6)
	Women	99.3 (98.8–99.9)	98.5 (97.5–99.6)	96.8 (95.1–98.5)	91.8 (88.5–95.0)	70.4 (63.4–77.4)
	Both	99.0 (98.4–99.5)	97.6 (96.4–98.7)	96.0 (94.4–97.6)	88.0 (84.7–91.3)	67.0 (60.5–73.4)
Hodgkin lymphoma						
	Men	91.3 (88.8–93.7)	77.6 (70.3–84.9)	72.5 (62.9–82.1)	62.7 (51.1–74.2)	45.4 (28.8–61.9)
	Women	95.6 (93.7–97.6)	92.8 (86.5–99.0)	76.7 (64.8–88.5)	54.9 (38.4–71.3)	28.2 (16.5–39.8)
	Both	93.2 (91.6–94.8)	82.5 (77.0–87.9)	73.9 (66.4–81.5)	60.2 (50.7–69.7)	36.0 (26.1–45.9)
Non-Hodgkin lymphoma						
	Men	77.4 (74.4–80.5)	74.0 (70.6–77.4)	75.8 (72.7–78.9)	63.1 (59.7–66.5)	42.5 (38.7–46.4)
	Women	83.9 (80.4–87.4)	85.9 (82.6–89.2)	78.5 (75.5–81.6)	73.4 (70.2–76.6)	44.4 (40.9–47.9)
	Both	79.8 (77.5–82.1)	78.8 (76.3–81.2)	77.0 (74.9–79.2)	67.7 (65.3–70.1)	43.5 (40.9–46.1)
Myeloma						

	Men	79.9 (68.9–90.9)	55.2 (46.4–64.0)	56.2 (50.4–61.9)	43.1 (38.1–48.1)	24.7 (20.3–29.0)
	Women	71.8 (57.0–86.6)	65.0 (55.8–74.1)	62.6 (56.6–68.7)	53.6 (48.4–58.8)	29.1 (24.9–33.4)
	Both	76.7 (67.8–85.7)	59.5 (53.1–65.9)	59.0 (54.9–63.2)	47.8 (44.2–51.4)	26.9 (23.9–30.0)
Acute lymphoid leukemia						
	Men	51.5 (41.0–62.0)	27.3 (10.0–44.5)	33.7 (14.1–53.3)	28.8 (6.3–51.4)	16.9 (1.5–32.4)
	Women	52.0 (38.6–65.5)	38.5 (18.4–58.7)	28.4 (9.6–47.3)	6.5 (-3.3–16.3)	13.9 (0.8–27.1)
	Both	51.7 (43.4–60.0)	32.6 (19.1–46.1)	31.2 (17.3–45.2)	17.6 (4.3–30.9)	14.8 (4.4–25.1)
Chronic lymphoid leukemia						
	Men	94.8 (83.9–105.7)	86.4 (77.7–95.0)	89.0 (84.3–93.7)	79.9 (74.6–85.2)	58.6 (51.6–65.7)
	Women	100.4 (100.4–100.4)	88.3 (79.4–97.2)	89.7 (84.1–95.2)	84.2 (78.5–90.0)	62.2 (55.5–68.8)
	Both	96.2 (88.1–104.4)	87.1 (80.8–93.4)	89.2 (85.6–92.9)	81.5 (77.5–85.5)	60.4 (55.5–65.3)
Acute myeloid leukemia						
	Men	53.6 (46.1–61.1)	45.8 (36.5–55.1)	27.1 (19.8–34.5)	11.1 (7.1–15.1)	1.8 (0.1–3.5)
	Women	64.9 (57.5–72.4)	50.8 (41.2–60.4)	33.6 (25.3–41.9)	20.1 (14.2–26.0)	2.4 (0.3–4.5)
	Both	59.0 (53.7–64.4)	48.3 (41.6–55.0)	30.1 (24.5–35.6)	14.8 (11.3–18.2)	2.1 (0.7–3.4)
Chronic myeloid leukemia						
	Men	95.5 (90.8–100.2)	97.4 (92.1–102.6)	88.6 (78.6–98.7)	67.4 (52.6–82.2)	36.0 (22.4–49.7)
	Women	93.6 (86.4–100.8)	89.1 (78.3–99.9)	87.4 (76.7–98.2)	80.0 (66.1–93.9)	43.0 (26.2–59.8)
	Both	94.8 (90.9–98.8)	94.6 (89.4–99.7)	88.1 (80.6–95.6)	73.1 (62.6–83.5)	39.0 (28.3–49.7)
Leukemia NOS and others						
	Men	70.6 (56.5–84.7)	81.5 (68.8–94.1)	58.4 (46.1–70.8)	40.5 (31.7–49.4)	20.7 (13.0–28.3)
	Women	61.4 (42.0–80.9)	66.0 (46.8–85.1)	46.2 (29.6–62.7)	44.9 (32.2–57.7)	20.9 (13.6–28.2)
	Both	67.2 (55.6–78.9)	75.8 (64.9–86.8)	53.8 (43.6–63.9)	41.8 (34.5–49.1)	20.7 (15.4–26.1)
All cancers[b]						
	Men	74.6 (73.7–75.6)	57.1 (56.3–57.9)	60.7 (60.1–61.2)	59.5 (59.0–60.0)	41.4 (40.8–42.0)
	Women	84.0 (83.4–84.7)	77.9 (77.3–78.5)	71.9 (71.3–72.6)	62.1 (61.4–62.8)	41.2 (40.5–42.0)
	Both	80.3 (79.7–80.8)	68.2 (67.7–68.7)	64.7 (64.3–65.1)	60.3 (59.9–60.7)	41.4 (40.9–41.8)

[a]The International Cancer Survival Standards (ICSS) age groups are used, which vary for prostate cancer. [b]The "all cancers" category excludes non-melanoma skin cancer. Abbreviations: NOS, not otherwise specified.
